# Attenuating ETEC virulence using a heat-labile enterotoxin–blocking binding protein

**DOI:** 10.1080/19490976.2025.2597567

**Published:** 2025-12-19

**Authors:** Marcus Petersson, Jens Sivkær Pettersen, Helena Bay Henriksen, Ágnes Duzs, Monica L. Fernández-Quintero, Nick Jean Burlet, Natalia Mojica, Ute Krengel, Timothy P. Jenkins, Andrew B. Ward, Thomas Emil Andersen, Jakob Møller-Jensen, Lone Gram, Andreas Hougaard Laustsen, Sandra Wingaard Thrane

**Affiliations:** a Department of Biotechnology and Biomedicine, Technical University of Denmark, Kgs. Lyngby, Denmark; b Bactolife A/S, Copenhagen, Denmark; c Department of Biochemistry and Molecular Biology, University of Southern Denmark, Odense, Denmark; d Department of Chemistry, University of Oslo, Oslo, Norway; e Department of Integrative Structural and Computational Biology, The Scripps Research Institute, La Jolla, USA; f Department of Clinical Microbiology, Odense University Hospitaland the Research Unit of Clinical Microbiology, University of Southern Denmark, Odense, Denmark

**Keywords:** Enterotoxigenic *Escherichia coli*, infection model, heat-labile enterotoxin, single-domain antibody, *Vibrio cholerae*, cholera toxin, binding proteins, gastrointestinal infections

## Abstract

Bacterial enteric pathogens are major contributors to the global burden of diarrheal diseases and the associated consequences for human health including malnutrition, growth stunting, morbidity, and mortality. While mortality from diarrhea has decreased, incidence remains high, and better interventions for preventing disease are needed. Single-domain antibodies (i.e., V_H_Hs), functioning as target-binding proteins in the gastrointestinal tract, have been proposed as a potential approach to mitigate bacterial pathogenesis. Here, we describe a mitigation strategy where precision binding of a bivalent V_H_H to the receptor-binding B-pentamer of heat-labile enterotoxin aggregates the AB_5_ toxin and impairs enterotoxigenic *Escherichia coli* colonization in a flow chamber model simulating the human intestine. The V_H_H construct also binds to the structurally similar cholera toxin and effectively abrogates its intestinal cell cytotoxicity in vitro. Based on these results, we believe that targeting virulence could emerge as a new strategy for the management of bacterial enteric pathogens, supporting gut health in at-risk populations alongside vaccination campaigns or in populations without access to vaccines.

## GRAPHICAL ABSTRACT

**Figure:**
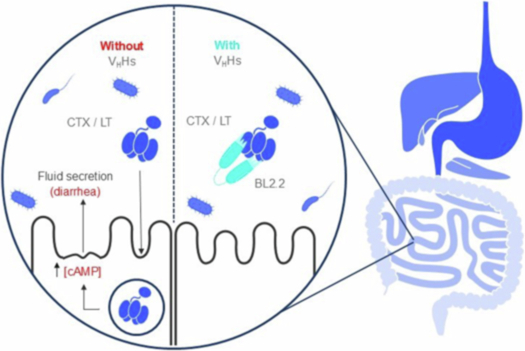

## Introduction

Diarrheal diseases kill more than one million people globally each year. They disproportionately affect low- and middle-income countries (LMICs) and are the second leading cause of death for children under five years of age.^1^^,^^2^ A major contributor to acute diarrhea in resource-limited settings is the food- and water-borne pathogenic bacterium enterotoxigenic *Escherichia coli* (ETEC).^3^^,^^4^ ETEC strains exhibit remarkable heterogeneity in their virulence factor repertoire, characterized by diverse combinations of heat-labile enterotoxin (LT), heat-stable toxins (STa and STb), and a wide array of colonization factors (CFAs).^5^ Further, *E. coli* exhibits a remarkable capacity for horizontal gene transfer, enabling a continuously evolving population of pathotypes.^6^^,^^7^ This diversity poses significant challenges in identifying key targets for the development of effective control strategies, such as vaccines, with protective effect and broad coverage.^8^ However, while ETEC colonization and infection are orchestrated by a sequential deployment of these virulence factors, effective delivery of toxins to epithelial cells remains essential to ETEC virulence.^9^^,^^10^

The pathogenic pathway of ETEC is similar to that of *Vibrio cholerae*, primarily due to their ability to produce two genetically and functionally related enterotoxins: LT from ETEC and cholera toxin (CTX) from *V. cholerae.*^10-12^ Phylogenetic analysis suggests that LT and CTX evolved from a common ancestor.^11^ This is reflected in their shared structural features as AB_5_-type toxins, the high sequence similarity between the receptor-binding B-subunits of LT (LTB) and CTX (CTXB) (83% sequence identity), and their common intestinal cell surface receptor, GM1, to which both toxins bind with strong (nanomolar) affinity.^13-16^ Both LTB and CTXB compromise intestinal barrier function, which enables toxin endocytosis and the intracellular release of the toxic A-subunit.^10^^,^^17^^,^^18^ The A-subunits of LT (LTA) and CTX (CTXA) then spur the activation of adenylate cyclase (via Gs_α_) and facilitate the opening of ion channels, which leads to ion efflux across the intestinal epithelium and ultimately secretory diarrhea driven by osmosis.^10^^,^^19^

Maternal antibodies (IgA and IgG) against LT and CTX found in breast milk confer protection against disease from both ETEC and *V. cholerae.*^18^^,^^20^ Similarly, we recently demonstrated that camelid-derived single-domain antibodies (i.e., V_H_Hs) can attenuate *V. cholerae* virulence by blocking CTX–GM1 binding.^21^ V_H_Hs retain the antigen affinity of full-length antibodies but demonstrate the high pH and protease resistance needed to effectively neutralize intestinal pathogens and their virulence factors in the gastrointestinal tract.^22-24^ Additionally, their small size and robust format enable efficient, low-cost production using industrially relevant microbial expression systems and biomanufacturing processes.^25^

In line with the above, a previous study has indicated that oral supplementation of the LT-neutralizing bivalent V_H_H BL2.2 can support a healthy and diverse gut microbiota of piglets exposed to ETEC, potentially mitigating post-weaning diarrhea.^26^ Here, we characterize BL2.2 for its ability to effectively abrogate LT-induced cytotoxicity in human intestinal cells. We characterize the interaction interface between the bivalent V_H_H construct and LT and the highly similar CTX, respectively, and reveal how natural GM1 receptor binding of both toxins is efficiently blocked by the bivalent V_H_H construct. Lastly, we show that this highly efficient blocking of LT significantly reduces the ability of human pathogenic ETEC strain H10407 (LT^+^ ST^+^) to colonize human Caco-2 cell layers in a flow chamber infection model and preserves epithelial integrity. Our findings thus indicate that neutralizing a single key virulence factor can effectively reduce bacterial pathogenesis, and that toxin-neutralizing V_H_H constructs could find utility as tools for limiting the global burden of diarrheal diseases.

## Materials and methods

### Commercially available materials and reagents

CTX (C8052), CTXB (C9903), and GM1 (G7641) were purchased from Sigma-Aldrich. Lyophilized CTX was dissolved in dH_2_O to a concentration of 0.5 mg ml^−1^. CTXB and GM1 were dissolved in phosphate-buffered saline (PBS, pH 7.4) to a concentration of 1 mg ml^−1^.

### Toxin expression and purification

For the molar ratios presented in this study, LTB and CTXB denote a single B-subunit (11.6 kDa) in LTB or CTXB pentamers (58 kDa). The holotoxins LT/CTX (85 kDa) include the catalytic subunit, LTA/CTXA. LT was expressed and purified as previously described.^27^ ETEC strain H10407 (LT^+^ ST^+^) was incubated in CAYE media (Merck, 100060) at 37 °C while shaking (200 rpm) for 18 hours. Both cell-bound LT and LT from the cell supernatant was extracted. The supernatant was separated by centrifugation at 4,000 × *g* for 10 min. The remaining cell pellet was dissolved in PBS and sonicated for 7 min using a Fisherbrand™ Model 120 Sonic Dismembrator (Fisher Scientific) to retrieve cell-bound LT. The sonicated cells were centrifugated at 15,000 × *g* for 20 min and the supernatant collected. Cell-bound LT and LT from the cell supernatant were pooled and purified using D-galactose resin (Thermo Scientific, 20372) as previously described.^28^

LTB-6HIS-3FLAG was produced by *E. coli* BL21 (DE3) using autoinduction medium (Formedium, AIMTB0205) supplemented with 50 µg ml^−1^ kanamycin at 30 °C, while shaking at 150 rpm for 20 hours. LTB was extracted from the periplasm and purified using immobilized metal affinity chromatography (IMAC) as previously explained.^21^ Purified LTB was dialyzed against PBS overnight before protein purity was assessed by SDS-PAGE.

For in vitro evaluation of BL2.1 and BL2.2 binding and toxin neutralization capacity, commercially available CTX (Sigma-Aldrich, C8052) and CTXB (Sigma-Aldrich, C9903) were used. For size-exclusion chromatography (SEC) analysis of BL2.1/BL2.2 and toxin interaction analysis, LT and CTX were produced in *Vibrio natriegens* as previously described.^29^ Briefly, cells harboring the LT or CTX plasmid were grown at 30 °C in LB-v2 salts medium (LB media supplemented with 204 mM NaCl, 4.2 mM KCl and 23.1 mM MgCl_2_) with chloramphenicol (25 μg ml^−1^). When cells reached an OD_600_ of ~0.8, L-arabinose was added to a final concentration of 0.2% to induce LT/CTX production. After 20 h, proteins were harvested from the culture media by two rounds of centrifugation at 8,500 × *g* for 30 min at 4 °C. The supernatant was filtered, loaded on a D-galactose column (Pierce, Thermo Scientific, 20372), and the protein was eluted using 50 mM Na-phosphate pH 7.4, 200 mM NaCl, 500 mM D-galactose. The LT- or CTX-containing fractions were pooled, concentrated and further purified by SEC using a Superdex 200 increase 10/30 GL column (Cytiva) pre-equilibrated with PBS.

### Toxin biotinylation

CTXB and LTB were mixed with No-Weigh NHS-PEG_4_-Biotin (Thermo Scientific, A39259) at a toxin:biotinylation reagent ratio of 1:2 and incubated at room temperature for 30 min. Excess reagent was reduced at least 125-fold by adding the mixture to 3 kDa molecular weight cut-off (MWCO) protein concentrators (Thermo Scientific, 16311964) together with PBS, followed by centrifugation at 4,300 × *g*.

### Plasmid construction and V_H_H dimerization

The V_H_H BL2.1 was originally discovered by Harmsen et al. (2009) and optimized for *E*. *coli* expression using the pSANG10-3F vector as previously described.^23^^,^^30^^,^^31^ The bivalent V_H_H construct BL2.2 was created by genetic fusion of two BL2.1 using a (GGGGS)_3_ linker as described elsewhere.^26^

### Expression and purification of V_H_H constructs

BL2.1 was produced by *E. coli* BL21 (DE3) using autoinduction medium (Formedium, AIMTB0205) supplemented with 50 µg ml^−1^ kanamycin at 30 °C, while shaking at 150 rpm for 20 hours. Cells were pelleted by centrifugation at 6,000 x *g* for 10 min and resuspended in periplasmic buffer (25% sucrose with 0.1% lysozyme in PBS). After 30 min incubation at room temperature, an equal amount of cold water was added to the solution and incubated on ice for 10 min. The periplasmic fraction was extracted by centrifugation at 20,000 x *g* for 30 min and purified using IMAC as previously described.^21^ The eluate was diluted 20 × in 25 mM Na–acetate (adjusted with HCl to a pH of 3.9) and further purified by cation exchange chromatography using a HiTrap SP FF 5 ml column connected to an NGC purification system (Bio-Rad).

BL2.2 was produced at Novonesis laboratories (Bagsværd, Denmark) using microbial fermentation with secretory expression of *N*-glycosylated protein. The supernatant containing BL2.2 was sterile-filtered and frozen before delivery.

BL3.2 was produced in *Komagataella phaffii* and purified using IMAC as previously described.^21^ In brief, an overnight culture (BMGY media) was used to inoculate BMMY media (OD_600_ = 1), which was incubated for 2 days at 30 °C and 160 rpm. Each day, the culture was supplemented with 1% methanol. Prior to purification, the media was centrifuged (15,000 x *g*, 15 min), and the supernatant collected. IMAC purification of the supernatant was carried out using 50 ml HisPur Ni-NTA resin and IMAC wash buffer at a 1:1 ratio (w:w), eluted using 20 ml IMAC elution buffer followed by 15 ml 1 M imidazole.

Protein concentration was determined using either a Qubit 4 fluorometer (Fisher Scientific, Q33238) and a broad range protein quantification kit (Fisher Scientific, A51292) or a NanoDrop One spectrophotometer (Fisher Scientific, ND-ONE-W). Protein purity was accounted for using visual assessment of SDS-PAGE analysis.

### Affinity determination using BLI and SPR

The kinetic parameters of *E. coli*-expressed, non-*N*-glycosylated BL2.1 were measured using both bio-layer interferometry (BLI) and surface plasmon resonance (SPR). For BLI experiments, an Octet RED96 instrument from ForteBio was used and data were analyzed as previously described.^21^ In short, biotinylated BL2.1 (76 nM) was captured by a streptavidin biosensor (Satorius, 18-5020). The biosensor coated with BL2.1 was then exposed to serial dilutions of four different concentrations (120–0.470 nM) of either CTXB or LTB. Kinetic parameters were determined using the Octet Analysis Studio software v12.2.2.26 (ForteBio).

A Biacore 8K+ GoldSeal (GE Healthcare Biosciences) was used for all SPR analysis. The kinetic interactions between BL2.1/BL2.2 and CTXB/LTB pentamers were analyzed both with BL2.1/BL2.2 as the ligand and CTXB/LTB as the analyte, and vice versa. Ligands were immobilized on a CM5 sensor chip (Cytiva, 29149603). All analytes were diluted in HBS-EP+ buffer (Cytiva, BR100669) and evaluated at a wide range of concentrations (1–300 nM) using single-cycle kinetics. Each analysis contained a startup phase of 120 s contact time and 60 s dissociation time with a flow rate of 30 µl min^−1^ at 25 °C. CTXB was analyzed using a contact time of 180 s and a dissociation time of 1000 s. LTB was analyzed using a contact time of 180 s 120 s and a dissociation time of 10000 s. BL2.1 was analyzed using a contact time of 30 s and a dissociation time of 7000 s. All analytes were evaluated at a flow rate of 30 µl min^−1^ at 25 °C. Data were analyzed by first subtracting the reference flow cell and HBS–EP+ buffer signals and fitting the data (excluding refractive index and baseline drift) with a global model (1:1 of binding sites). Kinetic parameters were determined using the Biacore Insight Evaluation Software v5.0.18.22102 (Cytiva).

### Determination of blocking capacity of toxin-receptor interaction

The ability of BL2.1 and BL2.2 to hinder the interaction between CTXB/LTB pentamers and the ganglioside GM1 receptor was determined using GM1 immobilized onto microplates in a previously described dissociation-enhanced lanthanide fluorescent immunoassay (DELFIA).^24^^,^^30^^,^^32^

60 µl of GM1 (Sigma-Aldrich, G7641) was directly immobilized (5 µg ml^−1^) onto 96-well Immuno Plates (Thermo Scientific, 437111) by incubation overnight. The plates were washed three times with PBS and blocked with PBS supplemented with 3% non-fat milk (M-PBS). BL2.1 and BL2.2 (36 nM) were mixed with biotinylated CTXB or LTB (36 nM) in M-PBS and incubated at 37 °C for 30 min before being added to the GM1-immobilized plate for 1 hour at room temperature. A biotinylated toxin-only (CTXB pentamer) control (36 nM) was included. The plates were washed again, three times with PBS-Tween (0.2%) and three times with PBS, and 100 ng ml^−1^ of streptavidin-conjugated europium (PerkinElmer, 1244-360) diluted in DELFIA assay buffer (PerkinElmer, 4002-0010) was added. Following 30 min incubation at room temperature, the previous washing procedure was repeated and europium fluorescence activated by adding DELFIA enhancement solution 20 (PerkinElmer, 4001-0010). Intensity measurements (excitation at 320 nm and emission at 615 nm) were carried out using a microplate reader (Victor Nivo) and the blocking capacity determined by relative comparison to the negative toxin-only control.

### Competitive binding assay

The ability of BL2.2 to bind the same epitope on CTXB as the previously reported V_H_H construct BL3.2 was assessed using a competitive binding DELFIA similar to the methods described above.^21^ In short, 60 µl of BL3.2 (3.2 µM) was directly immobilized onto 96-well Immuno Plates (Thermo Scientific, 437111). BL2.2 (360 nM) was mixed with biotinylated CTXB (36 nM) and added to the plate. A biotinylated toxin-only control (36 nM) was included as well. Fluorescence was detected by adding 100 ng ml^−1^ of streptavidin-conjugated europium (PerkinElmer, 1244-360) diluted in DELFIA assay buffer (PerkinElmer, 4002-0010) and DELFIA enhancement solution 20 (PerkinElmer, 4001-0010), followed by intensity measurements using a Victor Nivo microplate reader. The blocking capacity was determined by relative comparison to the negative toxin-only control.

### Detection of intracellular cAMP in cell-based assay

The ability of BL2.2 to neutralize the cytotoxic activity of CTX and LT was determined using a human HCA-7 colon cancer cell line (AddexBio, C0009003) as previously described.^21^ CTX (0.23 nM) or LT (4.60 nM) was incubated with different concentrations of either BL2.2 (0–62.5 nM and 0–78 nM, respectively) or a bivalent V_H_H construct without specificity for CTX or LT (57.5–230 nM and 1.15–4.60 µM, respectively) before being added to 1 × 10^4^ cells per well. BL2.2 was analyzed in duplicates of technical triplicates, the bivalent V_H_H control was analyzed once in replicates of six. The amount of intracellular cAMP was detected after 2 hours according to the manufacturer’s protocol (cAMP-Glo™ Max Assay, Promega, Madison, WI). Luminescence was measured using a Victor Nivo Multimode plate reader.

The cAMP standard curve was prepared in triplicates according to the manufacturer’s protocol (cAMP-Glo™ Max Assay, Promega, Madison, WI) and the effect of BL2.2 on the concentration of intracellular cAMP determined using a sigmoidal four parameter logistic regression model (R^2^ = 0.9745). The relative IC_50_ for BL2.2 was calculated based on a variable slope model. Neutralization capacity of BL2.2 against CTX and LT in HCA-7 cells was calculated relative to toxin-only and untreated (HCA-7) controls using the following equation:

$\mathrm{Neutralization capacity}\left(\%\right)=100\times \left(\frac{\left(\mathrm{BL}2.2\;+\mathrm{Toxin}\right)-\mathrm{Toxin}}{\mathrm{HCA}7\mathrm{untreated}-\mathrm{Toxin}}\right)$

### Structure prediction and characterization of the BL2.2-toxin interface

Artificial intelligence-based tools have revolutionized the field of protein structure prediction.^33-36^ In this study, ColabFold in combination with classical molecular dynamics (MD) simulations were used to predict and characterize the protein-protein interface of BL2.2 in complex with CTXB and LTB pentamers, respectively. Starting from the predicted complex structures, three repetitions of 1000 ns of classical MD simulations were performed, using the AMBER 24 simulation software containing the pmemd.cuda module.^37^ The structure models were placed into cubic water boxes of TIP3P water molecules with a minimum wall distance to the protein of 12 Å.^38-40^ Parameters for all simulations were derived from the AMBER force field 14SB.^41^ To neutralize the charges, we used uniform background charges.^42-44^ Each system was carefully equilibrated using a multistep equilibration protocol.^45^ Bonds involving hydrogen atoms were restrained using the SHAKE algorithm, allowing a timestep of 2.0 femtoseconds.^46^ System pressure was maintained at 1 bar by applying weak coupling to an external bath using the Berendsen algorithm.^47^ The Langevin Thermostat was utilized to keep the temperature at 300 K during the simulations.^48^

The obtained MD simulations for BL2.2 in complex with CTXB and LTB pentamers were used to calculate contacts using the GetContacts software.^49^ The analysis performed using GetContacts software enabled the computation of interactions within a single protein structure, as well as between different protein interfaces, while also monitoring the evolution of contacts throughout the simulation. In addition to quantifying the contacts of the different poses, CPPTRAJ was utilized for hierarchical agglomerative clustering analysis to identify the most representative binding poses.^50^

### Complex formation studies

Recombinant LT (15 µM) or CTX (68 µM) were mixed with V_H_H constructs at a 2:1 (BL2.1:toxin) or 1:1 (BL2.2:toxin) molar ratio, to equalize the number of V_H_H binding sites. Samples were analyzed by SEC on an equilibrated (PBS) Superdex 200 Increase 10/300 GL column (Cytiva) using the ÄKTA Pure system (GE Healthcare). Chromatograms were normalized to the maximum peak absorbance and compared to the SEC profiles of LT, CTX, BL2.2, or BL2.1 alone.

### Protein deglycosylation

BL2.2 and the previously characterized CTXB-specific bivalent V_H_H construct were diluted to 1 mg ml^−1^ in 0.1 M KH_2_PO_4_ (pH 5.5) and mixed with endoglycosidase H (Sigma-Aldrich, 324717) to a 100:1 volume ratio. The mixtures were incubated at 25 °C for 2 hours and deglycosylation confirmed by SDS-PAGE (Fig. S1).

### ETEC colonization in a Caco-2 intestinal flow chamber model

The intestinal epithelium colonization model was a modified version of a previously published model.^51^ Caco-2 cells (ATCC, HTB-37) were maintained in Dulbecco’s Modified Eagle’s Medium (Gibco, 31966-021) enriched with 20% heat-inactivated fetal bovine serum (HI-FBS) (Biowest, S181H) and 1% penicillin-streptomycin (Corning, 30-002-CI). Cells were passaged at 60-90% confluency and used for experiments between passages 25 and 50. All cultures were maintained at 5% CO_2_ and 37 °C. For flow chamber culturing, cells were seeded in collagen-coated µ-slides (Ibidi, 80606) at a density of 1.2 × 10⁵ cells cm^−2^ and flow chambers subsequently connected to silicon tubing and an Ismatec^TM^ IPC High Precision Multichannel Pump. Cells were allowed to settle and adhere for 4 hours before initiating flow at a rate of 15 µl min^−1^ (Shear-stress: 0.019 dyn cm^−2^). One day post-seeding (DPS), the flow rate was increased to 60 µl min^−1^, and at 2 DPS, a pulsatile flow was applied (140 µl min^−1^, Shear-stress: 0.178 dyn cm^−2^) for 1 minute ON and 9 minutes OFF to simulate the intermittent nature of peristaltic movement in the gut. At 6 DPS, the medium was replaced with serum-reduced medium (2% HI-FBS) without antibiotics. This was done with or without the addition of 2.2 µg ml^−1^ of BL2.2, and the cells were incubated for 30 minutes at flow rate of 140 µl min^−1^ prior to infection with ETEC H10407 or LT-deficient mutant H10407Δ*eltA* (jf570) for 24 hours.^52-54^

Inocula were prepared by suspending bacteria from agar plates in Hanks' Balanced Salt Solution (HBSS) (Gibco, 14025092) to an OD_600_ = 0.2, corresponding to an inoculum of approximately 1.3 × 10^8^ CFU ml^−1^. Inocula were introduced under a flow of 15 µl min^−1^ for 10 min, before shifting to sterile media with a pulsatile flow (90 µl min^−1^, 1 minute ON, 9 minutes OFF) for the remaining infection period. ETEC-infected cell layers were harvested using 0.25% trypsin-EDTA (Biowest, L0932) with the addition of 0.1% Triton X-100 (Sigma-Aldrich, X100), and plated on Luria Bertani (LB) agar plates for estimation of colony-forming units (CFUs).

For intestinal cell viability analysis, Caco-2 cell layers within the µ-slides were stained using the LIVE/DEAD™ Viability/Cytotoxicity Kit (Thermo Fisher Scientific, L3224) with a modified protocol. Briefly, the cells were washed with HBSS, stained with 2 µM Calcein-AM for 30 minutes, rinsed again with HBSS, and then fixed with formalin for 15 minutes. To visualize tight junctions, cell layers were washed with HBSS, fixed with 10% formalin (Sigma-Aldrich, HT5011) for 15 minutes, and rinsed with PBS. Permeabilization was performed using 0.1% Triton X-100 for 15 minutes, followed by additional PBS washes. Blocking was carried out for 60 minutes with 5% bovine serum albumin (BSA) (Sigma Aldrich, A9418). Cells were incubated for 3 hours at room temperature with 2 µg ml^−1^ Occludin Monoclonal Antibody Alexa Fluor 488 (Invitrogen, OC-3F10) in 1% BSA followed by counterstaining with 500 nM 4′,6-diamidino-2-phenylindole (DAPI) for 10 minutes (Sigma-Aldrich, D9542). Fluorescence microscopy was carried out on an Olympus BX51 fluorescence microscope, equipped with a 10x objective lens and using Olympus cellSens Dimensions software (version 1.7).

### Data processing and visualization

GraphPad Prism (version 10) and R (version 4.3.2) were used for figure generation and statistical analyzes. For CFU comparisons, one-way ANOVA with Tukey’s HSD post-hoc test was performed using the aov() and TukeyHSD() function from the R stats package (version 4.3.2) (***: *p* < 0.001, **: *p* < 0.01, *: *p* < 0.05). Average values and standard deviations were calculated after transforming the values to the figure scale illustrated. CLC Main Workbench version 23.0.2 was used for sequence analysis and alignment.

## Results

### The monovalent BL2.1 binds to LT and CTX with high affinity

When characterizing an antibody construct for its ability to displace the natural interaction between a toxin and its receptor, binding affinity is a critical parameter. LTB and CTXB pentamers have apparent affinities in the low nanomolar range (0.57 and 0.73 nM, respectively) for multivalent binding to the GM1 receptor, due to the avidity effect.^13^ BLI and SPR were used to determine the binding affinity of BL2.1, the monovalent counterpart of BL2.2, towards the LTB- and CTXB-pentamers. BLI measurements of immobilized monovalent V_H_H BL2.1 against LTB and CTXB pentamers demonstrated apparent affinities of <0.001 nM and 0.33 nM, respectively (Table S1 and Fig. S2). SPR analysis of the same ligand–analyte interactions (BL2.1–LTB/CTXB) yielded affinities (avidities) of 0.003 nM for LTB and 0.37 nM for CTXB pentamers, respectively (Table S1 and Fig. S3). SPR analysis of the opposite biomolecular interaction (i.e., LTB and CTXB pentamers as ligands), revealed a BL2.1 binding affinity of 0.26 nM towards LTB and 8.23 nM towards CTXB (Table S1 and Fig. S4).

### BL2.2 inhibits CTX functionality

**Figure 1.**
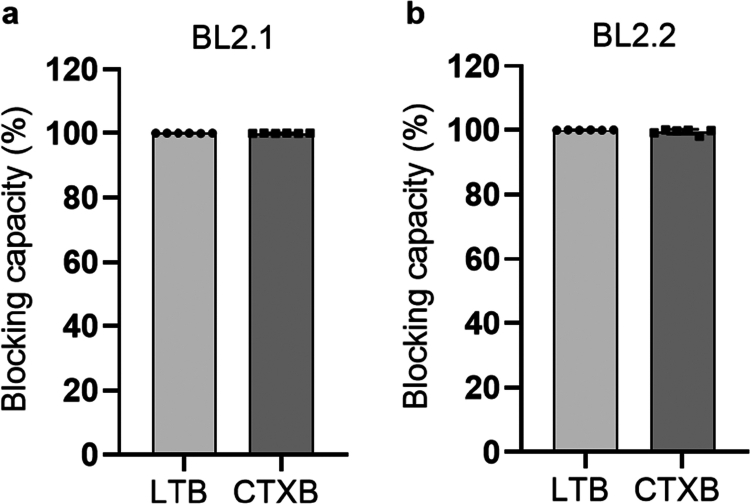
BL2.2 blocking capacity of LTB– and CTXB–GM1 interaction. (a) The ability of the monovalent V_H_H BL2.1 to abrogate the interaction between LTB and CTXB pentamers with the intestinal ganglioside receptor GM1 at a molar ratio of 1:1 (binding sites of LTB/CTXB:V_H_H). The average blocking capacity was calculated by normalization against a toxin-only control, after duplicate measurements of technical triplicates. Error bars indicate standard deviation. (b) The ability of V_H_H–V_H_H BL2.2, the bivalent counterpart of BL2.1, to block toxin (LTB or CTXB) interaction with GM1 at a molar ratio of 1:1 (LTB/CTXB:V_H_H binding sites). The average blocking capacity was calculated by normalization against a toxin-only control, after duplicate measurements of technical triplicates. Error bars indicate standard deviation.

Previous studies have shown that BL2.1 (i.e., LT109) can bind both LT and CTX, and hinder the LT-GM1 interaction.^30^ Additionally, its bivalent counterpart, BL2.2 (V_H_H–(GGGGS)_3_–V_H_H), has been shown to bind and neutralize LT from porcine ETEC strains in vivo.^26^^,^^55^ In a DELFIA, we specifically tested the in vitro CTXB-GM1 blocking capacity of BL2.1 and BL2.2. Both the monovalent BL2.1 and the bivalent BL2.2 blocked 100% of the interaction between CTXB and GM1 at a 1:1 CTXB:V_H_H/V_H_H–V_H_H molar ratio of binding sites, comparable to their blocking capacity (100%) of LTB–GM1 interaction at identical ratios (Figure 1). In contrast, a previously reported anti-CTX V_H_H selected based on target binding ability, demonstrated a CTXB–GM1 blocking capacity of 19% at a 1:100 toxin:V_H_H ratio without the ability to hinder LTB–GM1 interaction at the same concentration (Fig. S5).^56^^,^^57^ In the remainder of the study, the bivalent format of BL2.2 was employed to exploit avidity, given the multivalent nature of the LTB/CTXB pentamer.^21^^,^^58^^,^^59^

### BL2.2 neutralizes LT- and CTX-induced cytotoxicity

**Figure 2.**
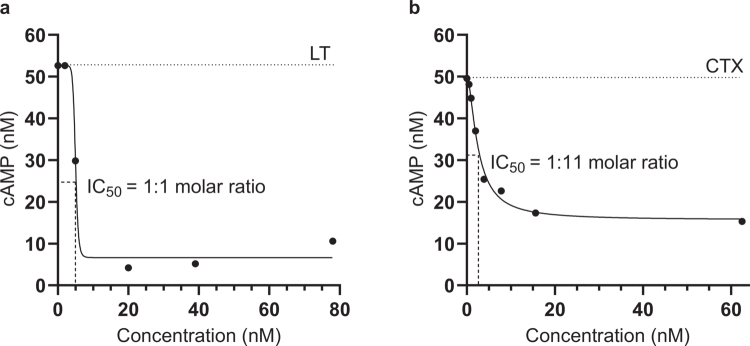
BL2.2 neutralization of LT and CTX toxicity in a human cell-based assay. (a) Neutralization of LT (4.60 nM) cytotoxicity in the presence of increasing concentrations (0–78 nM) of BL2.2. Levels of intracellular cAMP were interpolated from a sigmoidal four parameter logistic cAMP standard curve (R^2^ = 0.9745) based on triplicate measurements. Each data point represents an interpolated mean value from biological duplicates comprised of three technical replicates. (b) Neutralization of CTX (0.23 nM) cytotoxicity in the presence of increasing concentrations (0–62.5 nM) of BL2.2. Levels of intracellular cAMP were interpolated from a sigmoidal four parameter logistic cAMP standard curve (R^2^ = 0.9745) based on triplicate measurements. Each data point represents an interpolated mean value from biological duplicates comprised of three technical replicates.

Uptake of LT and CTX by endothelial cells of the small intestine induces increased levels of cellular cAMP.^18^^,^^60^ The toxin-neutralizing capacity of BL2.2 was assessed by adding increasing concentrations of BL2.2 (0–78 nM) to LT (4.60 nM) or 0–62.5 nM of BL2.2 to CTX (0.23 nM) and measuring the intracellular (HCA-7) cAMP concentration. BL2.2 reduced intracellular cAMP levels in HCA-7 intestinal epithelial cells seven-fold for LT and three-fold for CTX, relative to toxin alone (Figure 2). Normalized to this baseline, BL2.2 fully blocked cellular uptake of both LT and CTX at molar ratios of 1:2 and 1:135 (toxin:BL2.2), respectively (Fig. S6). This corresponds to a 5:4 (LT:BL2.2) and 5:270 (CTX:BL2.2) binding site ratio. By comparison, GM1-coated nanoparticles—used as receptor decoys to inhibit the CTX–GM1 interaction—achieved only a three-fold cAMP reduction in HCA-7.^60^ Notably, a bivalent V_H_H lacking toxin specificity showed no measurable impact on LT or CTX uptake (Fig. S6).

### BL2.2 is predicted to bind to the conserved GM1 binding site

**Figure 3.**
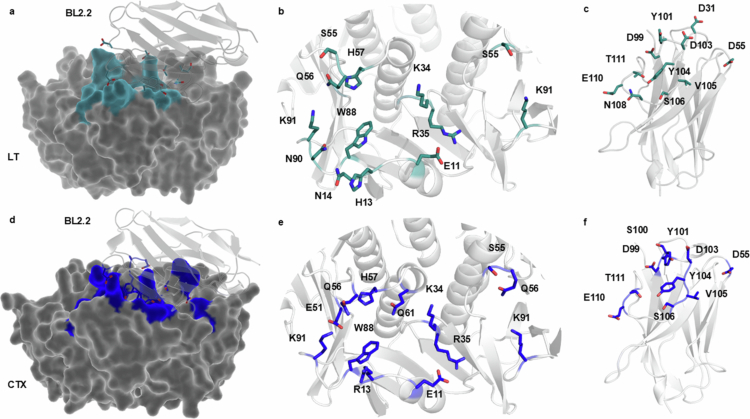
Prediction of the BL2.2 binding interface with LTB and CTXB pentamers. (a) The predicted protein-protein interface (blue) between BL2.2 (light gray) and LT (dark gray) based on ColabFold and molecular dynamics simulations. For visualization purposes, only one V_H_H domain of the bivalent BL2.2 molecule is shown. (b) The 11 amino acid residues (E11, H13, N14, K34, R35, S55, Q56, H57, W88, N90, and K91) in the LTB epitope predicted to be essential for BL2.2 binding, including two residues (S55 and K91) from the adjacent subunit in the pentamer. (c) The 11 amino acid residues (D31, D55, D99, Y101, D103, Y104, V105, S106, N108, E110, and T111) in the BL2.2 paratope predicted to be crucial for LT interaction. (d) The predicted protein–protein interface (cyan) between BL2.2 (light gray) and CTX (dark gray) based on ColabFold and molecular dynamics simulations. For visualization purposes, only one V_H_H domain of the bivalent BL2.2 molecule is shown. (e) The 11 amino acid residues (E11, R13, K34, R35, E51, S55, Q56, H57, Q61, W88, and K91) in the CTXB epitope predicted to be essential for BL2.2 binding, including three residues (S55, Q56, and K91) from the adjacent subunit in the pentamer. (f) The 10 amino acid residues (D55, D99, S100, Y101, D103, Y104, V105, S106, E110, and T111) in the BL2.2 paratope predicted to be important for CTXB pentamer interaction.

MD simulations were used to predict the most probable and stable complex formation between BL2.2 and both LT and CTX, respectively.^21^ Analysis of the protein–protein interface between BL2.2 and LT (Figure 3a) identified 11 distinct amino acids (E11, H13, N14, K34, R35, S55, Q56, H57, N90, W88, and K91) in the LTB epitope (Figure 3b). Among these residues, residue 13 is the only one that differs between LT and CTX in the GM1 binding site, with the exception of certain human LT (hLT) strains that carry an Arg at this position.^15^ Notably, S55 and K91 from two adjacent LTB subunits in the pentamer were predicted to contribute to the interaction. Ten amino acid residues (D31, D55, D99, D103, Y104, V105, S106, N108, E110, and T111) in the BL2.2 paratope are likely essential for LTB binding (Figure 3c). Analysis of the predicted BL2.2–CTX interaction (Figure 3d) revealed that out of the 11 amino acid residues (E11, R13, K34, R35, E51, S55, Q56, H57, Q61, W88, and K91) in the CTXB epitope, eight are shared by LTB (nine, if residue 13 is also considered) (Figure 3e). In this case, three residues (S55, Q56 and K91) contributed twice, from adjacent CTXB subunits. Similarly, eight out of the ten amino acid residues (D55, D99, S100, Y101, D103, Y104, V105, S106, E110, and T111) in the BL2.2 paratope important for CTXB binding, are shared between the two interactions (Figure 3f). Notably, the shared LTB/CTXB epitope of BL2.2 contains 9 of the 12 amino acids found in the CTXB epitope of BL3.2, a previously characterized V_H_H construct against CTX.^21^ Both BL2.2 and BL3.2 epitopes were predicted using equivalent MD simulations, with the BL3.2 epitope partially validated through hydrogen-deuterium exchange mass spectrometry (HDX-MS).^21^ In a competitive binding assay, BL2.2 prevented BL3.2 from binding CTXB at equimolar ratios (V_H_H construct:CTXB) (Fig. S7). The predicted BL2.2 epitope is in strong agreement with the established GM1 receptor-binding pocket of both LT and CTX, greatly conserved among hLTB and porcine LTB (pLTB), as well as the *ctxB* genotypes (*ctxB1*, *ctxB3*, and *ctxB7*) associated with all cholera pandemics to date (Fig. S8).^14^^,^^61^

### BL2.2 promotes toxin aggregation for enhanced neutralization

**Figure 4.**
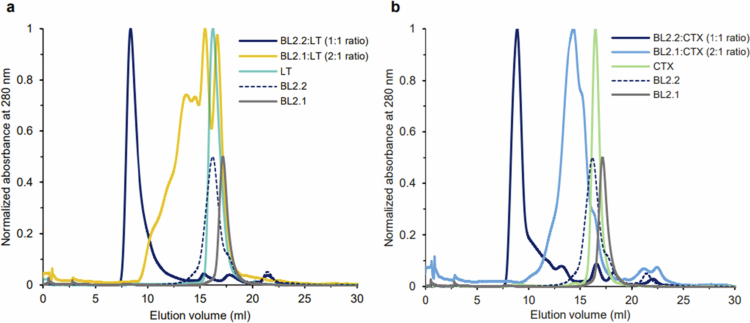
Size-exclusion chromatography (SEC) analysis of BL2.2 and BL2.1 complex formation with LT or CTX holotoxins. (a) Superimposition of SEC profiles of LT in complex with the bivalent V_H_H BL2.2 (dark blue) or its monovalent counterpart BL2.1 (yellow), with molar ratios indicated. For comparison, chromatograms of LT alone (cyan), BL2.2 (dashed blue line), and BL2.1 (gray) are also included. (b) Superimposition of SEC profiles of CTX in complex with the bivalent V_H_H BL2.2 (dark blue) or its monovalent counterpart BL2.1 (light blue). Profiles of CTX alone (green), BL2.2 (dashed blue line), and BL2.1 (gray) are also displayed. To aid in visual presentation, the absorbance of the V_H_H constructs alone was reduced to half in both panels.

The SEC profile of BL2.2 incubated with LT or CTX showed a peak eluting close to the void volume of the column, indicating that the bivalent V_H_H construct induced toxin aggregation (Figure 4a,b). In contrast, incubating the toxins with the monovalent BL2.1 at equivalent binding site ratios resulted in smaller protein complexes.

The original study of BL2.1 (i.e., LT109) reported that *N*-glycosylation enhances LT neutralization, potentially by increasing steric hindrance.^30^ Here, SPR analysis of LTB and CTXB pentamer interaction with glycosylated and non-glycosylated BL2.2 demonstrated that the *N*-glycans increased antigen avidity (Table 1, Fig. S9, and Fig. S10). *N*-glycosylated BL2.2 exhibited higher avidity towards LTB (0.27 nM) and CTXB (5.15 nM) pentamers compared to the non-glycosylated counterpart (1.25 nM and 8.55 nM, respectively).

**Table 1.** Comparison of *N*-glycosylated and non-glycosylated BL2.2 binding avidity towards LTB and CTXB pentamers determined using surface plasmon resonance (SPR).

	Glycosylated BL2.2			Non-glycosylated BL2.2		
Ligand	*k*_*on*_ (M^−1^ s^−1^)	*k*_*off*_ (s^−1^)	*K*_*D*_ (nM)	*k*_*on*_ (M^−1^ s^−1^)	*k*_*off*_ (s^−1^)	*K*_*D*_ (nM)
LTB pentamer	4.00 × 10^5^ ± 1.21 × 10^5^	0.96 × 10^−4^ ± 0.48 × 10^−4^	0.27 ± 0.20	2.84 × 10^5^ ± 0.43 × 10^5^	3.54 × 10^−4^ ± 0.02 × 10^−4^	1.25 ± 0.05
CTXB pentamer	1.18 × 10^5^ ± 0.09 × 10^5^	6.14 × 10^−4^ ± 0.44 × 10^−4^	5.15 ± 0.09	0.9 × 10^5^ ± 0.06 × 10^5^	8.03 × 10^−4^ ± 0.21 × 10^−6^	8.55 ± 0.03

The average equilibrium dissociation constant (*K*_D_) was calculated from duplicate measurements of the association rate constant (*k*_on)_ and dissociation rate constant (*k*_off)_. Standard deviation was used as a measurement of variation.

### BL2.2 abrogates ETEC virulence in a Caco-2 flow chamber infection model

LT has been reported to promote adhesion of ETEC to intestinal epithelial cells.^62^^,^^63^ In this study, an intestinal flow chamber infection model was utilized to evaluate the effect of LT neutralization by BL2.2 on ETEC virulence.^51^ Caco-2 cell layers were infected with either ETEC H10407 or an LT-deficient isogenic mutant (H10407Δ*eltA*), which exhibits reduced adhesion and impaired intestinal colonization.^62^^,^^64^ The LT-deficient mutant (H10407Δ*eltA*) was used to evaluate whether neutralization of the LTB pentamer by BL2.2 causes ETEC H10407 to behave as an LT-deficient strain.

**Figure 5.**
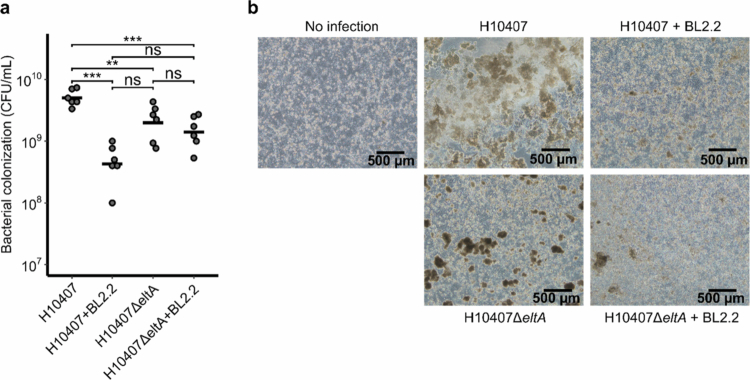
Suppression of ETEC colonization and virulence by BL2.2 in an intestinal flow chamber infection model. **(a)** CFU counts (CFU ml^−1^) after 24 hours infection in Caco-2 flow chambers: ETEC H10407 with BL2.2 (*p* = 4.2 × 10^−6^), the LT-deficient mutant H10407Δ*eltA* with BL2.2 (*p* = 1.4 × 10^−4^), and the LT-deficient mutant H10407Δ*eltA* without BL2.2 (*p* = 1.9 × 10^−3^), compared to ETEC H10407 alone. Statistical analyzes were performed using a one-way ANOVA with Tukey’s HSD post-hoc test (***: *p* < 0.001, **: *p* < 0.01, non-significant (ns): *p* > 0.05). (**b**) Phase contrast microscope images of infected Caco-2 cell layers, the same used for CFU counts in panel (**a**). Bacterial aggregates are visible on the surface of the Caco-2 cell layers.

CFU counts from infected cell layers revealed a significant (*p* = 4.2 × 10^−6^) 10-fold reduction in H10407 colonization after 24 hours when BL2.2 (2.21 µg ml^−1^) was added to the medium (Figure 5a). The impact of BL2.2 on ETEC colonization was more pronounced than the effect observed for the LT-deficient mutant (H10407Δ*eltA*), but this difference was not statistically significant (Figure 5a,b). BL2.2 did not significantly affect colonization by the LT-deficient mutant (H10407Δ*eltA*), supporting that BL2.2 neutralizes LT specifically. Tight junction integrity was compromised in H10407-infected cell layers, as evidenced by a more diffuse distribution of occludin staining, compared to layers co-incubated with BL2.2 or infected with the H10407Δ*eltA* mutant. Similarly, cell viability, assessed using Calcein-AM staining, was significantly reduced in H10407-infected layers (Fig. S11).

## Discussion

ETEC and *V. cholerae* are endemic to resource-limited settings, and their co-infection prevalence has been on the rise during the last decades.^65^ Oral cholera vaccines (OCVs) have been instrumental in combating cholera-induced diarrhea and Dukoral, which combines inactivated *V. cholerae* with 1 mg of recombinant CTXB, is the only licensed vaccine with a substantial effect (67% protection three months post-administration) also against ETEC diarrhea.^66-70^ Recent ETEC-vaccine development efforts have focused on combining CFA antigens with recombinant toxoids, such as LTB, CTXB, or LTB–CTXB hybrids to raise robust mucosal antibody responses and achieve broad strain coverage.^71^^,^^72^ However, while a broadly protective ETEC vaccine offers the potential for extensive protection, the associated higher development and implementation costs present significant barriers to accessibility and affordability in LMICs.^8^ Ultimately, effective population protection using enteric vaccines in resource-limited settings is complicated by poor distribution infrastructure, their impaired protection (e.g., due to environmental enteropathy), and a lack of financial incentives.^73-77^

Here, we evaluate a previously reported bivalent V_H_H construct BL2.2 based on its ability to neutralize both LT and CTX functionality in vitro and protect Caco-2 cells from ETEC (H10407 LT^+^ ST^+^) infection.^26^ BL2.2 was able to effectively hinder cellular uptake of both LT and CTX similar to a previously characterized V_H_H construct (i.e., BL3.2) that significantly decrease intestinal fluid secretion and burden of *V. cholerae.*^21^ Moreover, BL2.2 limited ETEC pathogenesis by significantly reducing bacterial colonization and preserving host cell viability and barrier integrity in an intestinal flow chamber model. This effect appears to be associated with the ability of BL2.2 to efficiently aggregate LT, preventing its interaction with host cells and thereby inhibiting the pathogenic effects of this toxin. Aggregation is likely induced by BL2.2 simultaneously engaging two B-pentamers, thereby cross-linking the toxins. This aligns with previous findings from other groups, which demonstrated that mismatched valency between toxins and inhibitors promotes aggregation and enhances potency.^78-80^ Moreover, the V_H_H construct can bind to two adjacent subunits in LTB or CTXB pentamers, which could further enhance the interaction.

The reduced colonization observed upon LT neutralization corroborates previous findings that LT promotes adherence of ETEC.^62^^,^^63^ In ETEC strains expressing F4 fimbriae, this mechanism is thought to involve the ADP-ribosylation activity of LTA, which elevates host intracellular cAMP levels, thereby stimulating the production of bacterial adhesins and promoting colonization.^63^ The extent to which this effect on adhesion involves modulation of the major intestinal adhesion factor CFA/I in H10407 remains to be determined. Yet, these findings suggest that the ability of BL2.2 to neutralize LT might be sufficient to limit the multifaceted intestinal pathogenicity of the prototypical LT^+^ ST^+^ ETEC strain responsible for human infection. However, while the intestinal flow chamber model effectively simulates physiologically relevant shear stress, which influences bacterial pathogenesis and stimulates epithelial development, it does not replicate key human host factors, such as immune cell interactions, host microbiota, and complex biochemical gradients.^81^^,^^82^ The absence of these factors should also be considered when evaluating the ability of BL2.2 to mitigate ETEC virulence.

Similar to previous studies on *V. cholerae* pathogenicity, our findings indicate that a V_H_H against LT or CTX can eliminate the role of these toxins in pathogenesis.^21^^,^^83^ The use of cross-protective antibodies against LT and CTX at the mucosal surface of the GI tract has been explored by several novel vaccines.^62^^,^^84-87^ Vaccines targeting conserved antigens (e.g., LT or highly conserved CFs) aim to stimulate heterotypic immunity, providing broad protection across diverse strains. However, mucosal antibody immunity in the gut tends to be relatively short-lived without frequent re-exposure.^85^^,^^88^ In our earlier study of piglets subjected to ETEC challenge, two oral administrations of 12.1 mg of BL2.2 were given per day and supported a diverse gut microbiota.^26^ Similarly, daily administrations of an anti-rotavirus V_H_H (35 mg kg^−1^) have been shown to reduce diarrheal onset in small children.^89^ V_H_H constructs possess several biophysical properties (e.g., high thermal stability and long-term storage stability) that could make them well-suited for diarrheal disease management in LMIC settings.^59^^,^^90^ Recently, the BL2.2 construct demonstrated robust thermal and processing stability, maintaining structural integrity and LT-binding activity after lyophilization and exposure to elevated temperatures (up to 95 °C) and high humidity.^55^ Such resilience supports its potential suitability for storage, transport, and deployment in LMIC settings, where exposure to elevated temperatures and lack of reliable cold chain infrastructure are common challenges.^91^

Furthermore, reported yields of V_H_Hs reaching 31 g L^−1^ in *Komagataella phaffii* or *Aspergillus niger* along with titers approaching 100 g L^−1^ for similarly sized proteins (cellulases) in *Trichoderma*, suggest that production costs can be driven well below $1 per day, assuming daily intake in the milligram range.^92-94^ This cost profile is highly favorable compared to conventional monoclonal antibodies ($95-200 per gram) and indicates that V_H_H constructs such as BL2.2 could provide affordable and continuous protection against enteric pathogens if consumed on a daily basis when at risk of infection.^23^^,^^26^^,^^95^

Half of all ETEC strains circulating in the human population produce LT, either alone or in combination with ST.^96^^,^^97^ The distribution of ETEC toxin profiles varies depending on geographical (e.g., seasonality and location) and host factors (e.g., age).^98^ Despite of the high diversity of ETEC strains found in patients, genomic analysis has inferred that ETEC-mediated diarrheal disease is most likely a set of overlapping global epidemics of individual ETEC lineages, which have been stable over considerable periods of time.^99^ This is deduced, as LT or ST gene alleles are linked to the chromosomal background. Toxin gene acquisition appears to provide a fitness advantage and remains key to virulence, as other *E. coli* strains can also acquire these genes to cause disease.^100-102^ In a comparable manner to LT, ST disrupt ion transport across intestinal epithelia, can cause watery diarrhea, and be pivotal to ETEC pathogenesis.^103^ Importantly, ST-only ETEC lineages are capable of causing severe diarrhea and the presence of both toxins can synergistically exacerbate diarrheal symptoms.^104-106^ The prevalence of ST-only strains and the phenotypic variation in toxin production emphasizes the need for evaluating BL2.2 against several clinically relevant strains to indicate its broadness of protection.^71^^,^^107^^,^^108^

LT and CTX both bind additional glycosylated structures, such as blood group antigens, beyond the GM1 ganglioside.^15^^,^^16^^,^^109^ In particular, fucosylation creates functional CTX receptors, providing evidence that these toxins can intoxicate intestinal cells through mechanisms that are not exclusively dependent on the GM1 ganglioside.^15^^,^^97^^,^^110-113^ Similarly, toxin binding to diverse glycoconjugates may explain why *N*-glycosylated BL2.2 binds more strongly to LTB and CTXB pentamers compared to deglycosylated BL2.2. In addition, glycosylation can enhance stability (e.g., protease resistance) with minimal safety risk for V_H_Hs confined to the GI tract, as demonstrated in long-term oral exposure studies.^114^^,^^115^

In addition to its role in toxin-mediated pathogenesis, prior studies have identified LT as a key factor in inducing enteropathic alterations on enterocyte surfaces and enhancing ETEC colonization.^18^^,^^116^ However, the combined downstream effects of these enterotoxins on a population level, such as their contribution to disease symptoms via secondary intestinal bindings sites or to childhood malnutrition, remain largely unexplored.^104^^,^^110^^,^^112^^,^^117^^,^^118^ Smaller recombinant antibody formats, such as V_H_Hs, can be selected using display technologies (e.g., phage display) to achieve high specificity and affinity for distinct epitopes, including toxins and cryptic or less accessible sites.^119^ We speculate that these properties could make V_H_Hs valuable tools for studying target-specific disease mechanisms, particularly those involving highly conserved virulence factors. Such investigations can provide insights into their roles in severe illness and their association with both short-term effects (e.g., diarrhea) and long-term outcomes (e.g., malnutrition and growth stunting).

While mortality from ETEC-associated diarrhea has decreased, incidence rate has not decreased comparably, and better interventions for preventing disease are needed.^120^ We suggest that targeted anti-virulence approaches might hold promise as new tools for managing bacterial enteric pathogens in the future, either as complementary strategies to vaccination programs or as standalone interventions for communities vulnerable to infection.

## Supplementary Material

Supplementary Material Supplementary_Information.docx

## Data Availability

The BL2.1 and BL2.2 protein sequence data used in this study are available in the Mendeley database [10.17632/bwsstxxtp2.1]. Source data are provided with this paper.
