# Supplementary material for: Attenuating ETEC virulence using a heat-labile enterotoxin–blocking binding protein
Source: Gut Microbes. 2025 Dec 19;18(1):2597567. doi: 10.1080/19490976.2025.2597567 (PMC12754688; doi:10.1080/19490976.2025.2597567)
Supplement: Supplementary Material — Supplementary_Information.docx [file KGMI_A_2597567_SM8766.docx]

**Supplementary Information**

**Table S1.** BL2.1 avidity towards LTB and CTXB pentamers.

| **Ligand** | **Analyte** | **Technology** | ***k_on_***  **(M^−1^s^−1^)** | ***k_off_***  **(s^−1^)** | ***K_D_***  **(nM)** |
| --- | --- | --- | --- | --- | --- |
| BL2.1 | LTB | BLI | 6.97 × 10^4^  ±  0.02 × 10^4^ | <1.0 × 10^-7 a^ | <0.001 |
| BL2.1 | CTXB | BLI | 1.08 × 10^5^  ±  1.84 × 10^2^ | 3.53 × 1^-5^  ±  0.09 × 10^-5^ | 0.33  ±  0.01 |
| BL2.1 | LTB | SPR | 7.93 × 10^6^  ±  1.10 × 10^6^ | 2.48 × 10^-5^  ±  1.20 × 10^-5^ | 0.003  ±  0.001 |
| BL2.1 | CTXB | SPR | 5.57 × 10^5^  ±  0.34 × 10^5^ | 2.12 × 10^-4^  ±  0.85 × 10^-4^ | 0.37  ±  0.13 |
| LTB | BL2.1 | SPR | 6.96 × 10^5^  ±  0.68 × 10^5^ | 1.82 × 10^-4^  ±  0.26 × 10^-4^ | 0.26  ±  0.01 |
| CTXB | BL2.1 | SPR | 2.18 × 10^5^  ±  0.09 × 10^5^ | 1.79 × 10^-3^  ±  0.04 × 10^-3^ | 8.23  ± 0.14 |

Kinetic parameters for BL2.1 interaction with LTB or CTXB pentamers determined using bio-layer interferometry (BLI) and surface plasmon resonance (SPR). The equilibrium dissociation constant (K_D_) was calculated from measurements of the association rate constant (k_on_) and dissociation rate constant (k_off_). ^a^ Value below limit of detection for the Octet RED96 instrument.


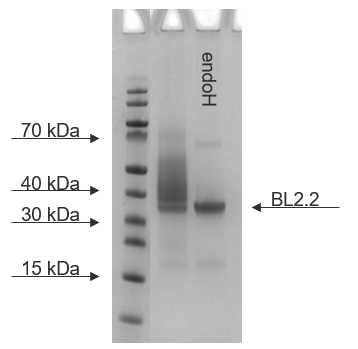


**Figure S1.** De-glycosylation of BL2.2 confirmed by SDS-PAGE. Comparison of untreated BL2.2 and BL2.2 treated with endoglycosidase H (endoH) in relation to a protein ladder.


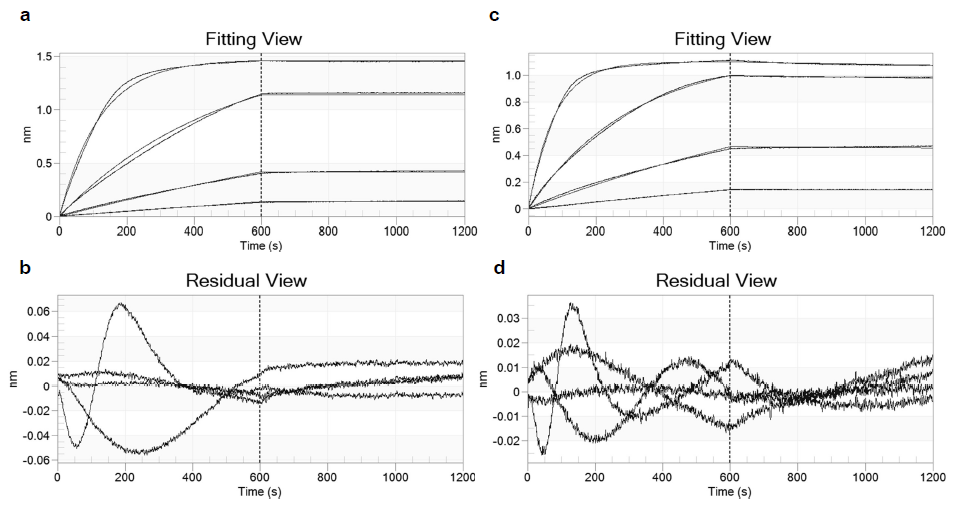


**Figure S2.** BLI sensorgrams for LTB and CTXB pentamers. (**a**) Sensorgram for the binding interaction between the ligand BL2.1 (76 nM) and the analyte LTB in a four-fold dilution series (120–0.470 nM). (**b**) The residual plot for BL2.1–LTB interaction, representing experimental data deviation from theoretical fitting. (**c**) Sensorgram for the binding interaction between the ligand BL2.1 (76 nM) and the analyte CTXB in a four-fold dilution series (120–0.470 nM). (**d**) The residual plot for BL2.1–CTXB interaction, representing experimental data deviation from theoretical fitting. The signal from the non-analyte control (LTB or CTXB) was subtracted from each sample and the data fitted to a global model (1:1 binding sites).

**
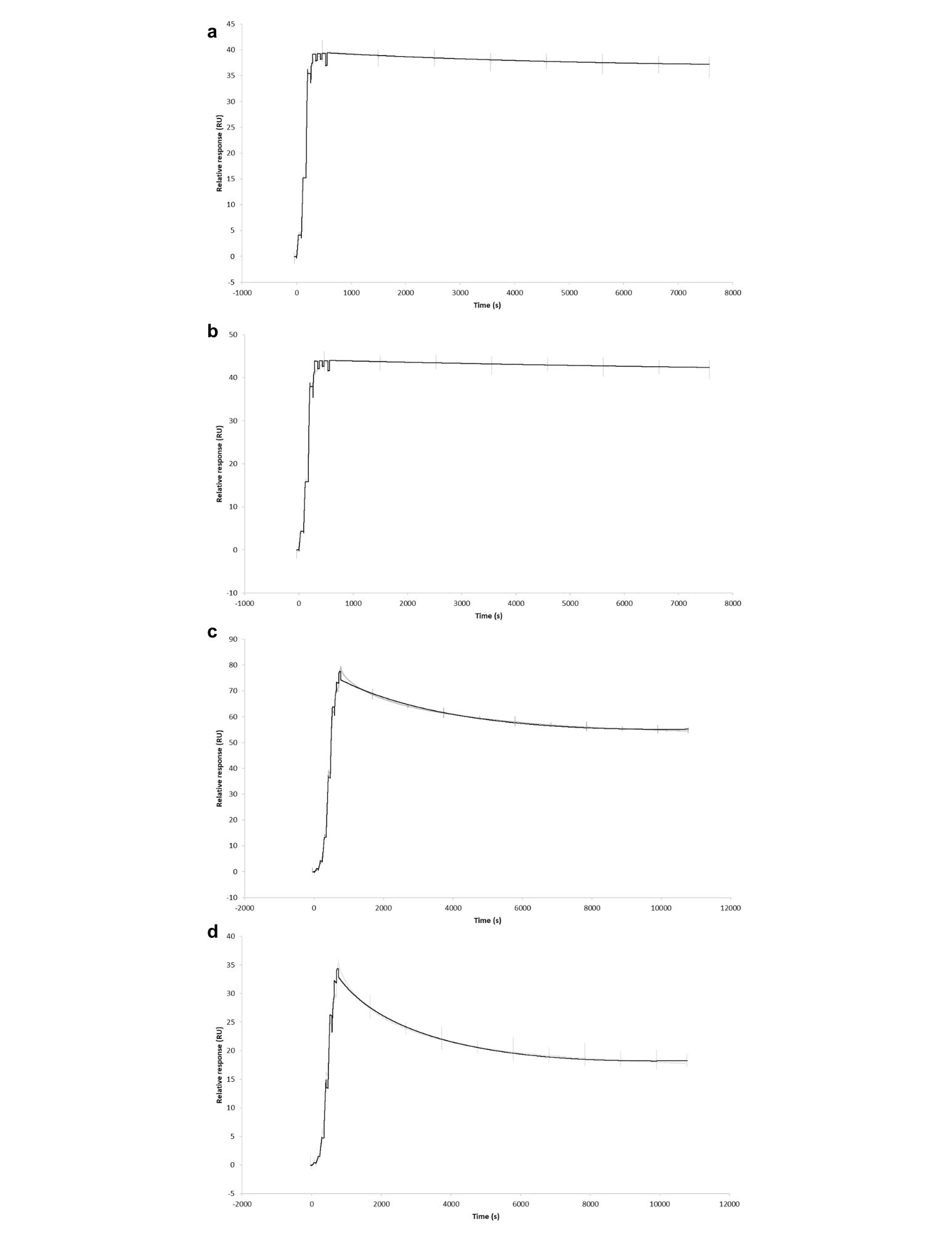
**

**Figure S3.** SPR sensorgrams for ligand-analyte interactions of BL2.1–LTB/CTXB. (**a,** **b**) SPR sensorgrams from duplicate measurements (single-cycle kinetics) of the biomolecular interaction between the ligand BL2.1 (1 µg ml^-1^) and analyte LTB (1–300 nM).

(**c**, **d**) SPR sensorgrams from duplicate measurements (single-cycle kinetics) of the biomolecular interaction between the ligand BL2.1 (1 µg ml^-1^) and analyte CTXB (1–300 nM).

**
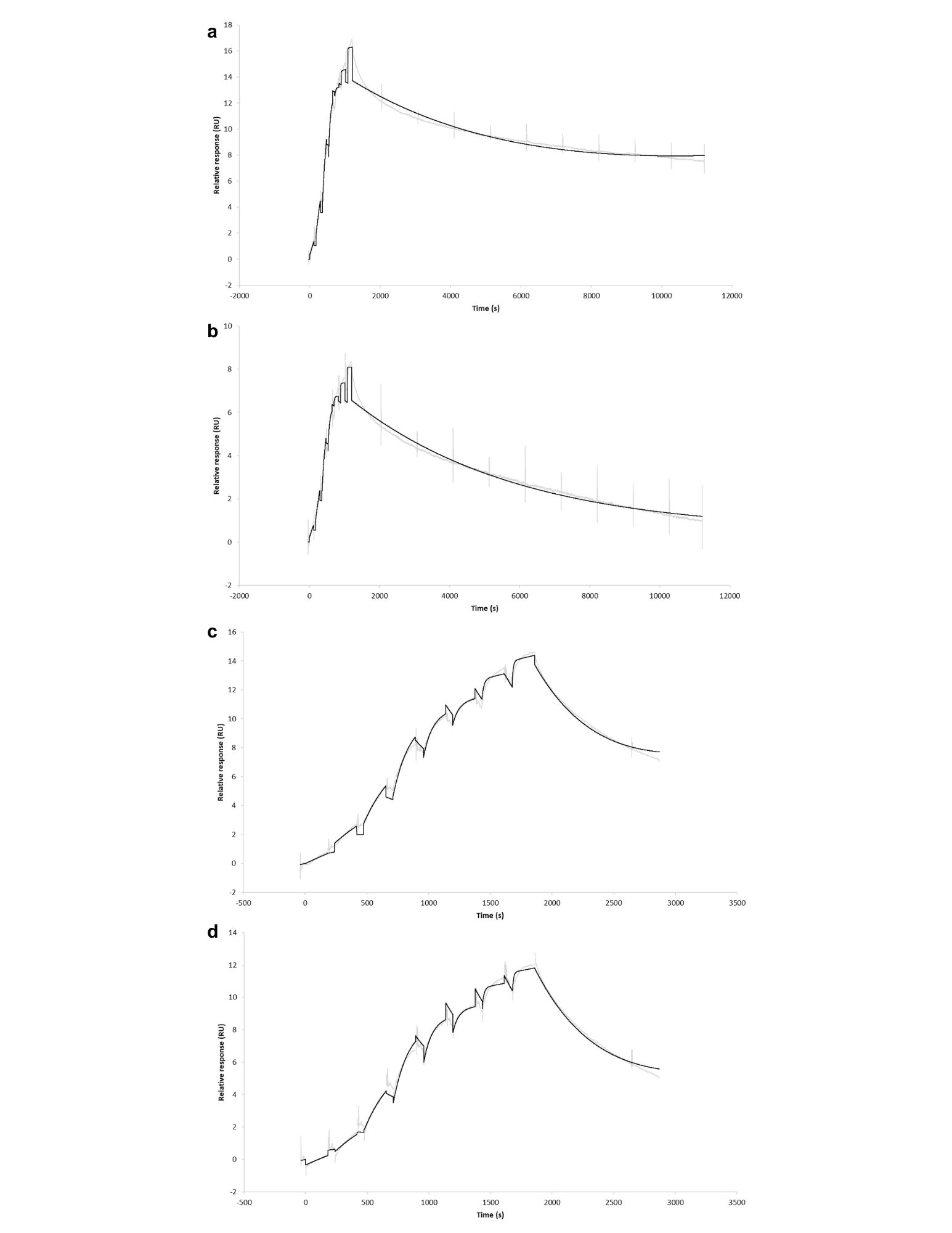
**

**Figure S4.** SPR sensorgrams for ligand–analyte interactions of LTB/CTXB–BL2.1. (**a,** **b**) SPR sensorgrams from duplicate measurements (single-cycle kinetics) of the biomolecular interaction between the ligand LTB (5 µg ml^-1^) and analyte BL2.1 (1-300 nM).

(**c,** **d**) SPR sensorgrams from duplicate measurements (single-cycle kinetics) of the biomolecular interaction between the ligand CTXB (5 µg ml^-1^) and analyte BL2.1 (1–300 nM).

**Figure S5.** Comparison of LTB– and CTXB–GM1 cross-blocking capacity of BL2.1 and an anti-CTX V_H_H construct. A comparison of BL2.1 and a previously reported anti-CTX V_H_H construct based on their blocking capacity of LTB and CTXB interaction with the GM1 ganglioside receptor at various V_H_H:toxin molar ratios (of binding sites)^56^. A similar V_H_H without target specificity was included as a control. The average blocking capacity was calculated by normalization against a toxin-only control. The of LTB– and CTXB–GM1 blocking capacity of BL2.1 was measured once in technical triplicates, whereas the anti-CTX V_H_H construct and the V_H_H control was analyzed in duplicate measurements of technical triplicates. Error bars represent standard deviation.

**Figure S6.** BL2.2 blocks intestinal cell uptake of LT and CTX. (**a**) The ability of BL2.2 (8.20–4.60 nM) to neutralise the functionality of LT (4.60 nM) measured by bioluminescent detection of intracellular cAMP in HCA-7 cells. Each bar represents the average blocking capacity from duplicate measurements of technical triplicates, normalized against a toxin-only control. A bivalent V_H_H construct (1.15–4.60 µM) lacking specificity for LT was included as a control and measured in six replicates, normalized against a toxin-only control. Error bars represent standard deviation. (**b**) The ability of BL2.2 (31.05–0.23 nM) to neutralise the functionality of CTX (0.23 nM) measured by bioluminescent detection of intracellular cAMP in HCA-7 cells. Each bar represents the average blocking capacity from duplicate measurements of technical triplicates, normalized against a toxin-only control. A bivalent V_H_H construct (57.5–230 nM) lacking specificity for CTX was included as a control and measured in six replicates, normalized against a toxin-only control. Error bars represent standard deviation.

**Figure S7.** Competitive binding assay for the GM1 receptor-binding epitope of CTXB. The ability of the previously reported bivalent V_H_H construct (BL3.2), which targets the GM1 receptor-binding site of CTXB, was assessed for CTXB that had been pre-incubated with BL2.2^21^. The average binding capacity was calculated by normalization against the BL3.2–CTXB only interaction from a single measurement of four technical replicates. Error bars indicate standard deviation.


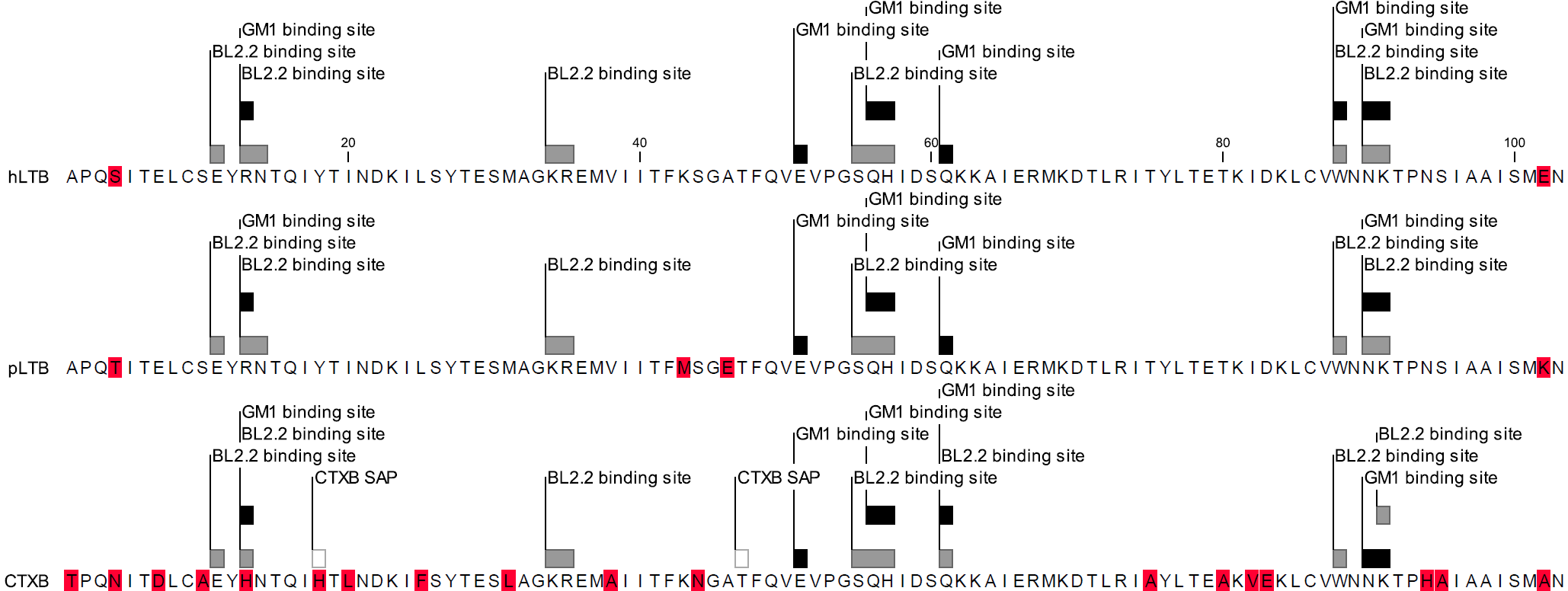


**Figure S8.** Amino acid sequence comparison of LTB from human (hLTB) or porcine (pLTB) isolates, as well as CTXB (*ctxB7*), including the predicted epitope for BL2.2 (light grey) based on machine learning simulations in this present study. The amino acid differences between each (hLTB, pLTB, and CTXB) toxin B-subunit is indicated in red, and the respective GM1 binding site in black^14^. The two sites (in white) for CTXB single amino acid polymorphism (SAP) represent the variability between all *ctxB* genotypes (*ctxB1*, *ctxB3*, and *ctxB7*) responsible for cholera pandemics to date^61^.


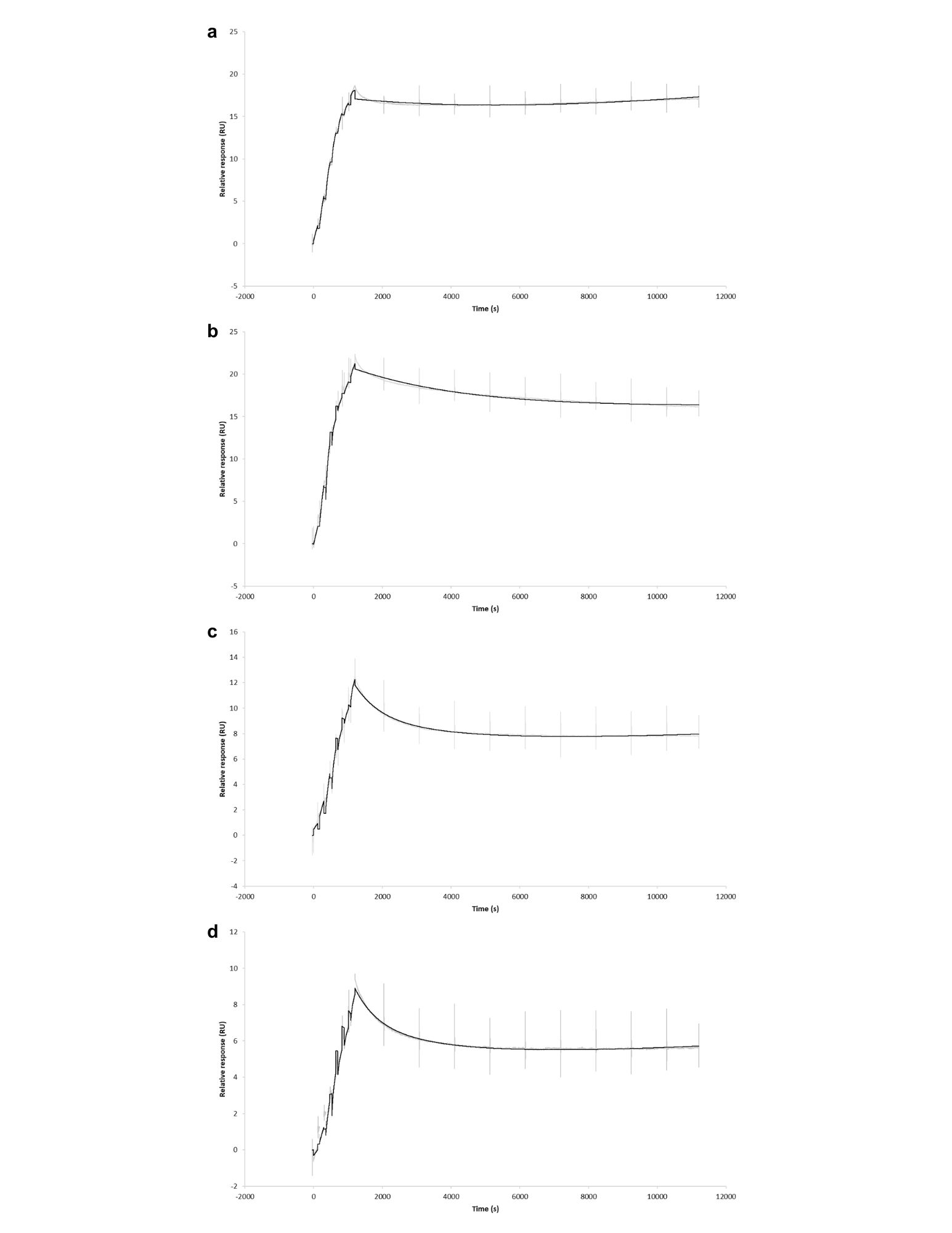


**Figure S9**. Surface plasmon resonance (SPR) sensorgrams for ligand–glycosylated analyte interactions of LTB–BL2.2 or CTXB–BL2.2. (**a,** **b**) SPR sensorgrams from duplicate measurements (single-cycle kinetics) of the biomolecular interaction between the ligand LTB (5 µg ml^-1^) and glycosylated analyte BL2.2 (1–300 nM).

(**c**, **d**) SPR sensorgrams from duplicate measurements (single-cycle kinetics) of the biomolecular interaction between the ligand CTXB (3 µg ml^-1^) and glycosylated analyte BL2.2 (1–300 nM).


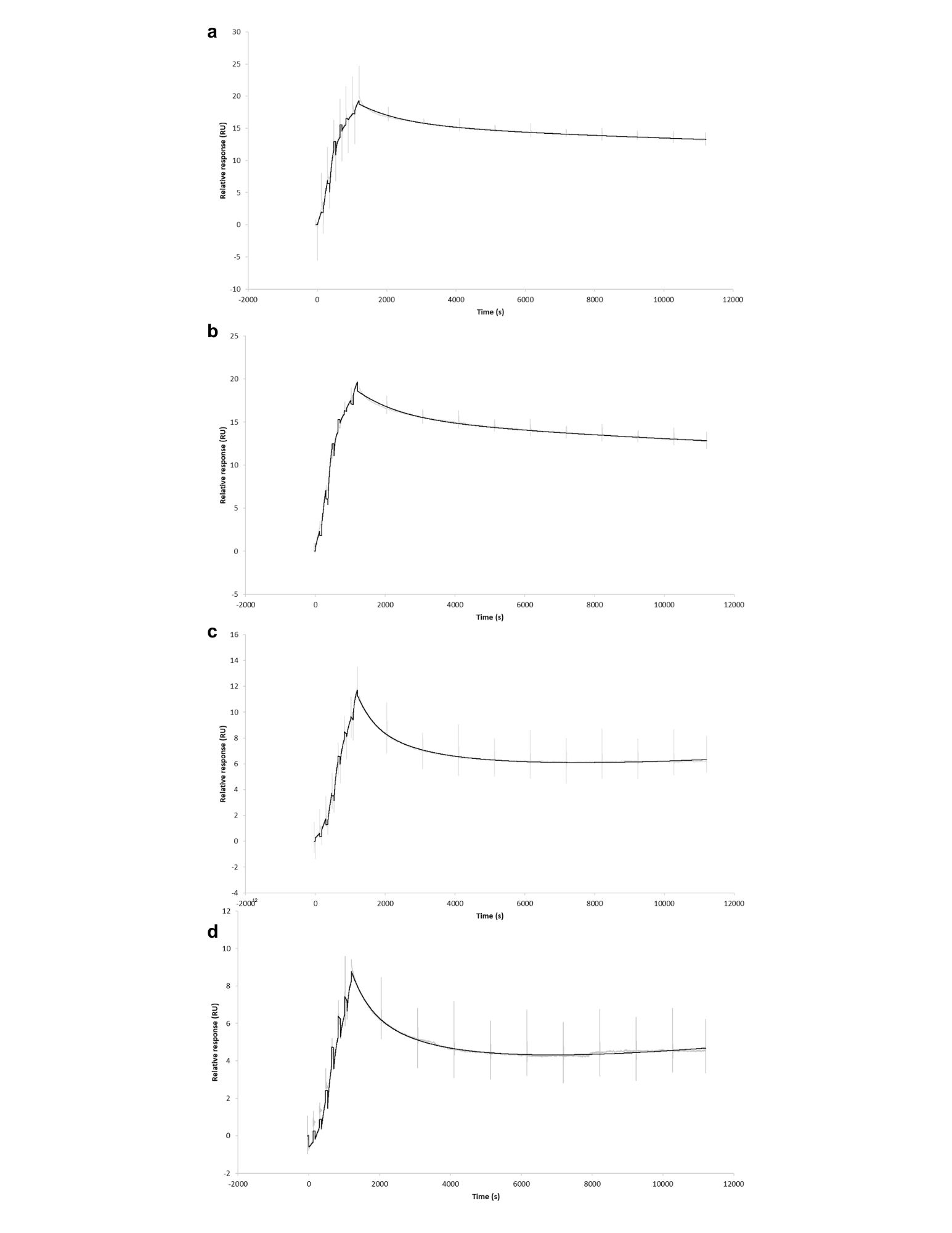


**Figure S10.** Surface plasmon resonance (SPR) sensorgrams for ligand–analyte interactions of LTB–BL2.2 or CTXB–BL2.2. (**a,** **b**) SPR sensorgrams from duplicate measurements (single-cycle kinetics) of the biomolecular interaction between the ligand LTB (5 µg ml^-1^) and non-glycosylated analyte BL2.2 (1–300 nM). (**c**, **d**) SPR sensorgrams from duplicate measurements (single-cycle kinetics) of the biomolecular interaction between the ligand CTXB (3 µg ml^-1^) and non-glycosylated analyte BL2.2 (1–300 nM).


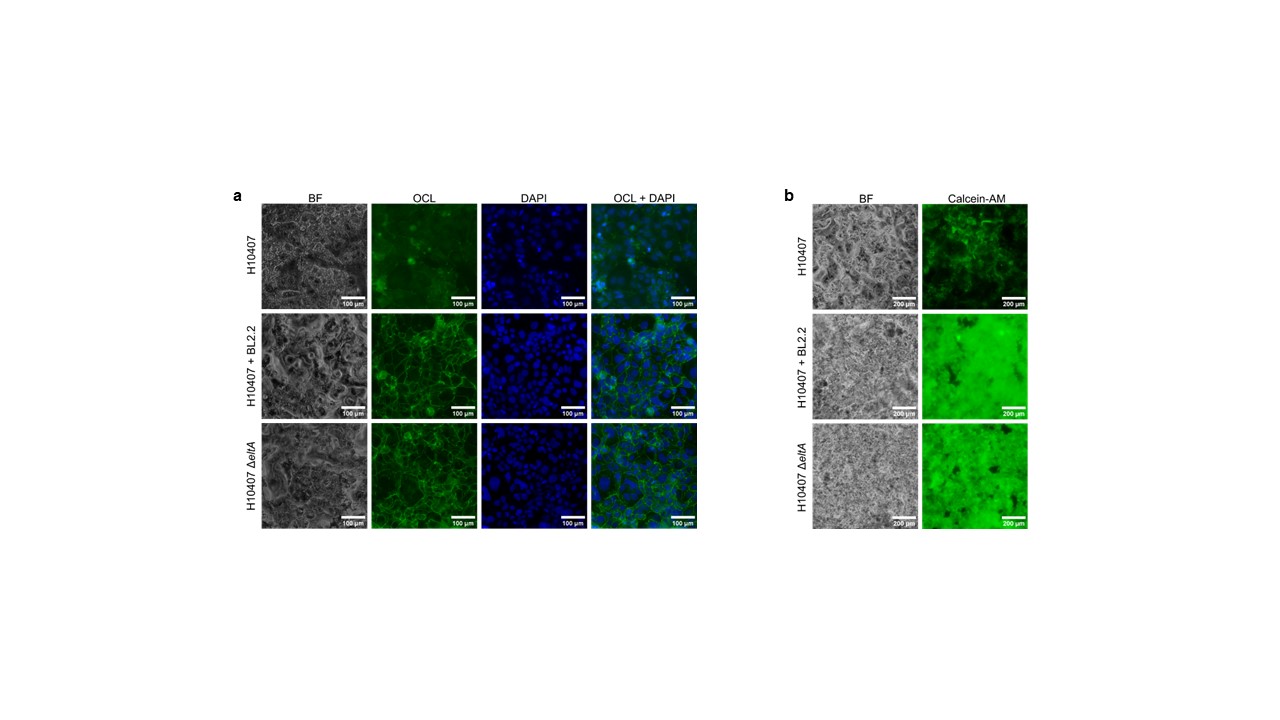


**Figure S11.** Viability and tight junction integrity of flow chamber-grown Caco-2 cell layers infected with H10407. **(a)** Tight junction protein (occluding, OCL) staining 18 hours after infection, with or without the addition of either BL2.2 or the LT-deficient H10407Δ*eltA*. Nuclei stained with 4′,6-diamidino-2-phenylindole (DAPI). **(b)** Bright-field (BF) and fluorescent (Calcein-AM) microscopy images of cell viability 18 hours after infection, with or without the addition of either BL2.2 or the LT-deficient H10407Δ*eltA.*
